# Contribution of Oswaldo Paulo Forattini to public health: analysis of scientific production

**DOI:** 10.1590/S1518-8787.2016050000217

**Published:** 2016-11-24

**Authors:** Juliana Gonçalves Reis, Keilla Miki Kobayashi, Helene Mariko Ueno, Cristiane Martins Ribeiro, Telma Abdalla de Oliveira Cardoso

**Affiliations:** I Programa de Pós-Graduação Saúde Materno-infantil. Faculdade de Medicina. Universidade Federal Fluminense. Niterói, RJ, Brasil; II Escola Politécnica de Saúde Joaquim Venâncio. Fundação Oswaldo Cruz. Rio de Janeiro, RJ, Brasil; III Programa de Pós-Graduação em Sustentabilidade. Escola de Artes, Ciências e Humanidades. Universidade de São Paulo. São Paulo, SP, Brasil; IV Núcleo de Biossegurança. Escola Nacional Saúde Pública. Fundação Oswaldo Cruz. Rio de Janeiro, RJ, Brasil

**Keywords:** Scientific and Technical Activities, Entomology, Scientific Communication and Diffusion, Bibliometrics, Historical Article

## Abstract

**OBJECTIVE:**

To analyze the main characteristics of the scientific production of Oswaldo Paulo Forattini, researcher and, for 40 years, editor of *Revista de Saúde Pública*.

**METHODS:**

Descriptive study with bibliometric approach conducted in three steps. (1) identification of bibliographic records using the following search strategy: “Oswaldo Paulo Forattini” OR “Forattini OP” OR “Forattini” up information sources Google Scholar, Web of Science, and PubMed, in July 2016, which retrieved 867 records. (2) composition of research corpus, in which we included 351 bibliographic records of articles, books, book chapters, editorials, book reviews, informative notes and annual reports of the RSP and excluded 516 duplicates and acknowledgement notes, obituary notes, and nonretrievable citations. (3) data organization and analysis, in which we built databases for descriptive analysis and development of the MeSH coauthors and terms networks in VOSviewer software. For analysis of editorials, three reviewers read the full text of each editorial and categorized them according to subject, historical context and perspectives, relating them with historical milestones.

**RESULTS:**

Forattini’s scientific production occurred from 1946 to 2009, most consisting of articles (n = 218; 62.1%), editorials (n = 43; 12.3%), and books (n = 13; 3.7%). The main subjects were *Culicidae* (36.8%), *Triatominae* (12.5%), and Epidemiology (10.0%). The coauthors of articles were his professors, colleagues of his generation, and graduate students. His editorials addressed critical reflections on the production of knowledge, research priorities, and factors that contributed to or hindered progress. The scope of subjects is broad, referring to socioeconomic and scientific development, public health issues in developed countries, or global health.

**CONCLUSIONS:**

The analysis shows Forattini’s commitment with public health, research with vectors, training of researchers, and scientific communication.

## RESUMO

**OBJETIVO:**

Analisar as principais características da produção científica de Oswaldo Paulo Forattini, pesquisador e, por 40 anos, editor da Revista de Saúde Pública.

**MÉTODOS:**

Estudo descritivo com abordagem bibliométrica realizado em três etapas. (1) Identificação dos registros bibliográficos, utilizando a seguinte estratégia de busca: “Oswaldo Paulo Forattini” OR “Forattini OP” OR “Forattini O” nas fontes de informação *Google Scholar*, *Web of Science* e PubMed*,* em julho de 2016, o que recuperou 867 registros. (2) Composição do *corpus* da pesquisa, na qual foram incluídos 351 registros bibliográficos de artigos, livros, capítulos de livros, editoriais, resenhas de livros, notas informativas e relatórios anuais da RSP e excluídos 516 duplicatas e notas de agradecimento, notas de obituários e citações não recuperáveis. (3) Organização e análise dos dados, na qual foram construídos bancos de dados para análise descritiva e elaboração das redes de coautores e de termos do MeSH no *software VOSviewe*r. Para análise dos editoriais, três revisores leram o texto completo de cada editorial e os categorizaram segundo assunto, contexto histórico e perspectivas, relacionando-o com marcos históricos.

**RESULTADOS:**

A produção científica de Forattini ocorreu de 1946 a 2009, a maioria composta por artigos (n = 218; 62,1%), editoriais (n = 43; 12,3%) e livros (n = 13; 3,7%). Os principais assuntos foram Culicidae (36,8%), Triatominae (12,5%) e Epidemiologia (10,0%). Os coautores dos artigos foram seus mestres, colegas de sua geração e alunos de pós-graduação. Seus editoriais abordaram reflexões críticas sobre a produção de conhecimento, prioridades em pesquisa e fatores que contribuíam ou desfavoreciam o progresso. O escopo dos assuntos é amplo, remetendo ao desenvolvimento científico e socioeconômico, questões de saúde pública em países desenvolvidos ou saúde global.

**CONCLUSÕES:**

A análise mostra o comprometimento de Forattini com a saúde pública, na pesquisa com vetores, na formação de pesquisadores e na comunicação científica.

## INTRODUCTION

To celebrate the 50 years anniversary of the *Revista de Saúde Pública* (RSP), we reviewed the scientific production of Oswaldo Paulo Forattini. His publications and academic history have been reported in other articles^13^ ^,^ ^15^ ^,^ ^16^. Forattini graduated in Medicine from the Universidade de São Paulo (USP) in 1949, was a professor of the Medical Entomology Program of the Faculdade de Saúde Pública of the Universidade de São Paulo (FSP-USP), and editor of the RSP for 40 years.

The RSP, published since 1967 by the FSP-USP replacing the Arquivos da Faculdade de Higiene e Saúde Pública, is established as one of the main scientific journals of Brazil. Its mission is to publish original contributions on relevant issues in public health, in Portuguese, English, and Spanish. Its scope is national and international, since its first volume published with indexing: Medline, PubMed, Thomson Reuters/ISI, Web of Science, Social Sciences Citation Index, Current Contents/Social Behavioral Science, Global Health, Biosis, Embase, HEALSAFE, POPline, Tropical Diseases Bulletin, Bulletin of Communicable Abstracts, Nutrition Abstracts & Reviews, and Wildlife Worldwide.

Considering the relevance of the RSP and Forattini’s activities in Public health, this study presents reflections on the relations between his careers as researcher and editor and the construction and development of the scientific field. According to Bourdieu (1989), a field emerges from practices of agents that constitute it, while the practices define and inhibit the possible conducts of the agents^1^. Hayashi (2014) points out that the social dimensions of scientific activity, from the perspectives of the Sociology of Science and Social Studies of Science, establish relations with the theoretical framework of Bibliometrics and Scientometrics^12^.

With this article we aimed to analyze the main characteristics of the scientific production of Oswaldo Paulo Forattini, researcher and, for 40 years, editor of the *Revista de Saúde Pública*.

## METHODS

This is a descriptive study with Bibliometric approach. According to Hayashi^12^ (2014, p. 302), “Bibliometrics and Scientometrics provide instruments to map and extract useful information to understand the social and intellectual structure of scientific fields and the social, economic and political aspects involved in scientific activity.”

**Figure 1:**
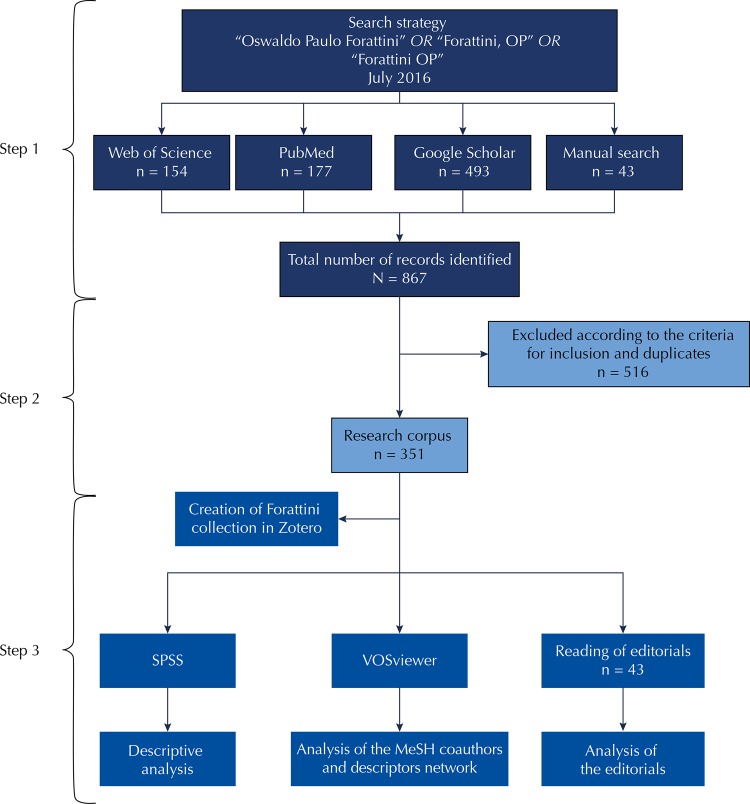
Study steps flowchart*.

SPSS: Statistical Package for the Social Sciences; MeSH: Medical Subject Headings

The survey was conducted in three steps: (1) identification of bibliographic records; (2) composition of research corpus; (3) data organization and analysis (Figure 1).

* Each step is represented by a tone of blue.

For step 1 – identification of bibliographic records – data collection was performed on the following information sources: Google Scholar, by creating the researcher’s profile in Scholar Google, Web of Science, and PubMed. Scholar Google presents greater scope in information retrieval. In addition to the peer-reviewed scientific literature, other types of documents can be identified, such as books and book chapters, as well as the number of citations. However, thorough curation is required to obtain quality data. The Web of Science is a multidisciplinary information source with higher quality bibliographic elements and PubMed is an open-access, healthcare-specific information source.

The search strategy employed is presented in Figure 1. Manual search for editorials published in the RSP was also conducted. To identify all bibliographic records, there was no restriction concerning year of publication.

In step 2, composition of research corpus, we excluded duplicates, acknowledgement notes, nonretrievable citations, obituary notes, and editorials in coauthorship and included the bibliographic records of articles, books, book chapters, editorials, book reviews, informative notes, and annual reports of the RSP.

In step 3, data organization and analysis, we created a collection of bibliographic records identified in the reference manager Zotero^a^. Two databases were built: the first in the Statistical Package for the Social Sciences (SPSS) for descriptive analysis; the second for preparation of infographics of the coauthors and terms networks of the Medical Subject Headings (MeSH) in VOSviewer software of the the Centre for Science and Technology Studies (CWST).

Three reviewers analyzed the RSP editorials by fully reading each editorial signed by Forattini. The content was categorized according to subject, historical context, and perspectives and was related to historical milestones.

## RESULTS AND DISCUSSION

The results are presented in table and pictures with their respective discussions. Until the completion of this article, on August 22, 2016, there were 6,840 citations retrieved in Google Scholar^b^.

**Table:** Historical series of scientific production of Oswaldo Paulo Forattini, second decade (1946-2009).

Variable	Historical series															

	1946-1955		1956-1965		1966-1975		1976-1985		1986-1995		1996-2005		2006-2009		Total	
							
	n	%	n	%	n	%	n	%	n	%	n	%	n	%	N	%
Documents identified	37	11.0	54	15.0	41	12.0	56	16.0	85	24.0	72	20.0	6	2.0	351	100
Type of document																
Articles	29	78.4	45	83.3	24	58.5	29	51.8	45	52.9	42	58.3	6	100	220	62.7
Editorial	-	-	-	-	-	-	9	16.1	17	20.0	17	23.6	-	-	43	12.3
Book	-	-	3	5.6	1	2.4	1	1.8	1	1.2	7	9.7	-	-	13	3.7
Book chapter	-	-	-	-	-	-	2	3.6	1	1.2	-	-	-	-	3	0.9
Notes	8	21.6	6	11.1	9	22.0	5	8.9	5	5.9	6	8.3	-	-	39	11.1
Book review	-	-	-	-	7	17.1	8	14.3	14	16.5	-	-	-	-	29	8.3
Event Annals	-	-	-	-	-	-	1	1.8	-	-	-	-	-	-	1	0.3
RSP reports	-	-	-	-	-	-	1	1.8	2	2.4	-	-	-	-	3	0.9
Subject																
Arbovirus	-	-	2	3.7	1	2.4	-	-	-	-	1	1.4	-	-	4	1.1
Ceratopogonidae	9	24.3	14	25.9	-	-	-	-	-	-	-	-	-	-	23	6.6
Culicidae	2	5.4	22	40.7	9	22.0	5	8.9	42	49.4	44	61.1	5	83.3	129	36.8
Ecology	-	-	-	-	-	-	1	1.8	3	3.5	-	-	-	-	4	1.1
Epidemiology	-	-	1	1.9	-	-	11	19.6	14	16.5	9	12.5	-	-	35	10.0
Leishmaniasis	4	10.8	6	11.1	5	12.2	1	1.8	-	-	-	-	-	-	16	4.6
Phlebotominae	5	13.5	4	7.4	5	12.2	2	3.6	-	-	-	-	-	-	16	4.6
Journal	-	-	-	-	-	-	5	8.9	6	7.1	2	2.8	-	-	13	3.7
Triatominae	2	5.4	1	1.9	14	34.1	26	46.4	-	-	-	-	1	16.7	44	12.5
Others	15	40.5	4	7.4	7	17.1	5	8.9	20	23.5	16	22.2	-	-	67	19.1

Forattini’s scientific production spanned six decades, the most fruitful from 1986 to 1995 with 24.0% of total documents. From 1946 to 1955, he published 37 articles, the first ones still as undergraduate student. He was the only author in five of the 14 works published as a student, showing his profile as a pathological and clinical researcher.

From 1948, he began to publish in Entomology and, gradually, his publications were predominantly – but not exclusively – in this area. In the 1950s, researches emerged on taxonomy of *Diptera* and *Triatominae* in general and research on Bionomics of vectors or potential vectors of infectious and parasitic diseases. Often, the two lines supported description or redescription of vector species.

Noteworthy, he collaborated with the Special Health Service of Araraquara in 1949–1950, in the region of Ribeirão Preto, São Paulo, to clarify the possible existence of autochthonous schistosomiasis mansoni. In these researches, he found autochthonous focus of Chagas disease and leishmaniasis.

From 1950 to 1960, he studied leishmaniasis in the São Paulo state as a member of the Committee for the Study of Leishmaniasis of the State Department of Public Health and Social Assistance of São Paulo, but also in the states of Mato Grosso and Paraná, in addition to the then territories of Amapá and Rondônia. These studies were an opportunity to associate medical entomology studies with the epidemiological context of transmission of infectious and parasitic diseases by using field research.

Thus, Forattini experienced contexts of disease transmission, determining transmission foci, reservoirs, potential vectors, and human behavior aspects that would justify such epidemiological situation. He continued the laboratory studies both on clinical findings in humans or reservoirs and on vector taxonomy and infectivity.

Based on these laboratory and field researches, Forattini published a book series called Entomologia Médica^2–5^ (1962; 1965a, 1965b, 1973), assumed as a specialty in Public Health and required for the study the epidemiology of diseases transmitted or determined by arthropods. His books addressed classification and systematics of the groups (richly illustrated by himself), biological aspects, and epidemiology and prophylaxis of diseases involving arthropods. His Entomology books are essential for students and field researchers. In these books he described several identification keys for species of *diptera* and *Triatominae* of interest in public health, which he designed with freehand drawing and ink in illustrative boards.

From 1976 to 1985, Forattini continued with research on leishmaniasis and *Phlebotominae*. In the same period, however, the encephalitis epidemic by Rocio virus in the Vale do Ribeira, São Paulo, led him to the region, where he began to study the potential vectors of this emerging arbovirosis. Again, the epidemiological characteristics of the transmission and of the people affected led him to formulate hypotheses about mosquito vectors and about the role of man in the increased risk of transmission of arboviruses. These hypotheses were tested until the end of his career.

In the context of the Brazilian epidemiological transition, the emergence of modern diseases (for example, chronic degenerative diseases, occupational and traffic accidents) caused no change to his focus on researching the epidemiology of infectious and parasitic diseases, with emphasis on environmental factors – natural and anthropogenic – that favored the transmission of these diseases, including proliferation, adaptation, and dispersal of vector populations. On the contrary, his experience in research reinforced that the environmental changes caused by humans inserted them in cycles of transmission of zoonoses or approached domiciled populations – those that benefited from the conditions offered by man, now living in the environment modified by humans.

The outbreak of dengue fever in the 1980s influenced Forattini’s studies and research group, leading to increased production on the subject in the following decades. From 1990 to 2005 several studies were published about *Culicidae* ecology, especially those of anthropic environment.

Forattini recognized the importance of books as a source of scientific information and, as editor, considered the RSP’s reviews section as an important medium to disseminate such material. Thus, throughout his career, such reviews accounted for 8.0% of his total production.

In his academic life, more than 200 articles and 14 books were published. His researcher career included studies on medical Entomology, from taxonomic aspects and general biology of diptera to studies on the distribution of these insects and their relation with the epidemiology of diseases transmitted by them. Therefore, no less important were his books “Epidemiologia Geral”^6^ ^,^ ^7^ – adopted as reference in the training of health professionals in the country – and “Ecologia, epidemiologia e sociedade”^8^, texts that supported the discipline “Ecology and Public Health.” Ahead of his time, in 2005 he published the book: “Conceitos básicos em epidemiologia molecular”^10^. In addition to high level of knowledge in these specific fields, the book “O ser e o ser humano”^9^ presents his philosophical reflections about human nature.

In a sociological mapping study, Santos et al.^17^, in 2016, assessed publications of the RSP from 1967 to 1977. The authors qualified as “gray box” articles on “experiments with animals, basic biological studies, microbiology, zoology, parasitology, entomology, taxonomy, animal physiology, and nutrition” (p. 419) and questioned why half of the articles address these subjects. That shows lack of knowledge as to Forattini’s profile as researcher and to the scope he gave to the RSP, as he valued basic science as the foundation for the country’s scientific development, as observed in the analysis of editorials presented below. Particularly in the period, the RSP was important as one of the few scientific journals on public health in the country. As Forattini was one of the most productive researchers and the FSP-USP was a reference in epidemiology of infectious and parasitic diseases, such expertise was expected to be represented in the RSP. In fact, the articles of his series on vector ecology and arbovirus epidemiology are the most cited.

**Figure 2:**
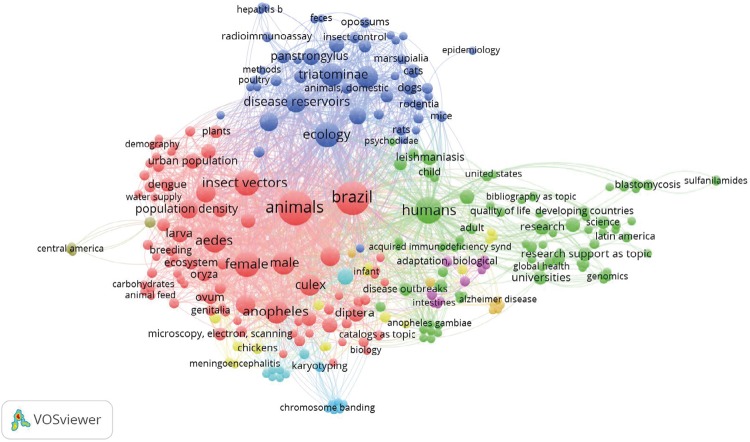
Infographic of subjects of articles by Oswaldo Paulo Forattini (1946-2009), 2016*.

Figure 2 represents the volume and the ratio of MeSH terms collected in publications, in which three thematic clusters are observed. In dark blue we present the production of the 1950s about clinical cases and works with *Triatominae* in the region of Araraquara, relating their findings with the anthropic influence in the wild environment. At that time, based on field research, relations were established between ecology, public health, and epidemiology. At the same time, the epidemiology of tegumentary leishmaniasis was studied in states other than São Paulo, investigating vectors and reservoirs. From 1959, he starts to focus deeper on mosquitoes of the family *Culicidae*, initially focusing on malaria vectors, but later extending to arbovirus, especially on the occasion of the epidemic caused by Rocio virus in the Vale do Ribeira region. In the early 1980s, a base was built for the field research activities in the city of Pariquera-Acu (Vale do Ribeira, São Paulo). Maintained until today, this base has trained technicians, laboratory equipped with magnifying glasses and other equipment for creation, sorting, storage, and identification of the material collected in the field, in addition to accommodation for researchers. This base was used in the 1990s and 2000s for the development of the series of studies with *Culicidae*, represented by the red cluster in Figure 2.

*The smaller and more dispersed circles (for example, light blue, orange, pink) represent terms cited with low frequency and in isolation, such as *chromosome banding*, *meningoencephalitis* and *alzheimer’s disease*, among others.

Forattini studied the domiciliation of the *Triatominae* and made important observations on the behavior and biology of these insects and their relations with the Protozoan parasite. He described dozens of new species of culicoides, reviews of species of *Culicidae* and published textbooks about *Culicidae* and other Diptera, used by entomologists as reference.

The consecutive thematic projects on ecology of Culicidae, led by him, resulted in dozens of articles and mentorship for master’s and doctoral degrees. He also acquired, by means of thematic project resources, a scanning electron microscope, used to observe eggs and anatomical structures of mosquitoes (as female cibarius), which enabled better accuracy in identifying species.

The green cluster in Figure 2 represents the editorials of his authorship, published in the RSP and in other journals of the area.

**Figure 3:**
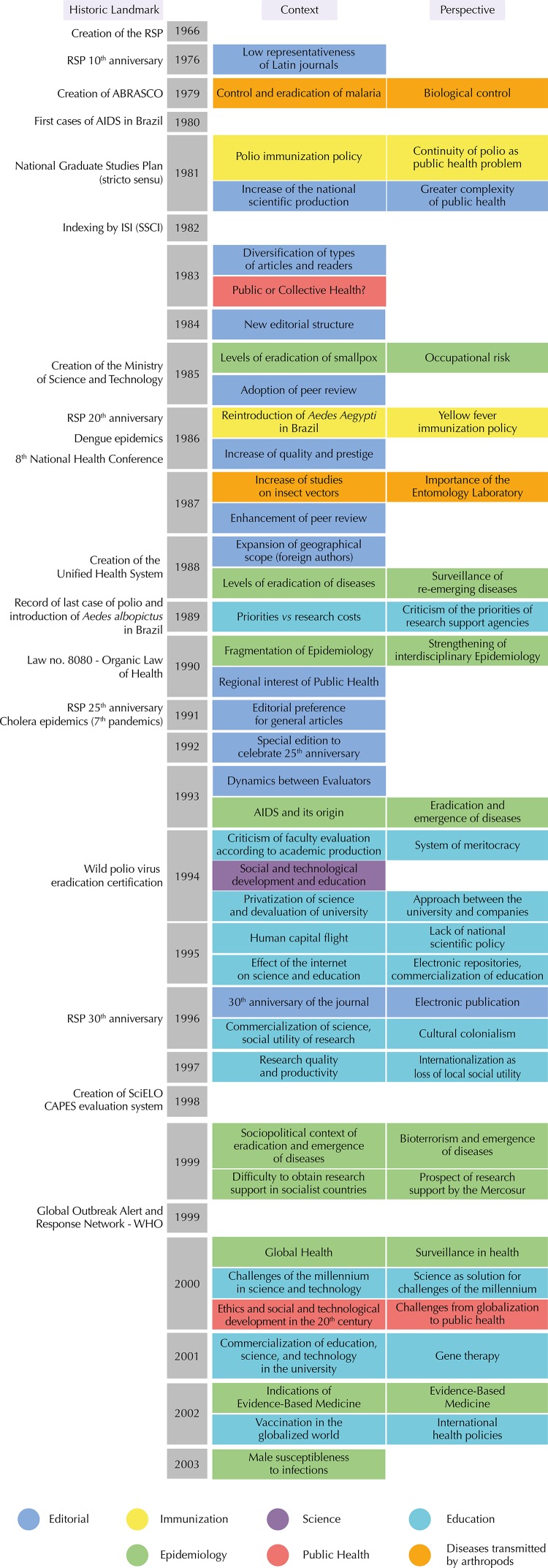
Summary of the analysis of editorials published by Oswaldo Paulo Forattini (1976-2003), 2016.

RSP: Revista de Saúde Pública; Abrasco: *Associação Brasileira de Pós-graduação em Saúde Coletiva*; ISI: Information Sciences Institute; SSCI: Social Sciences Citation Index; Capes: *Coordenação de Aperfeiçoamento de Pessoal de Nível Superior*; OMS: *Organização Mundial da Saúde*; Abrasco: *Associação Brasileira de Saúde Coletiva*; ISI (SSCI): Institute for Scientific Informationm (Social Sciences Citation Index)

Figure 3 shows a summary of the content analysis of his editorials. It is observed that the scope of subjects increased over time, referring to socioeconomic and scientific development, public health issues in developed countries, or global health. Such editorials go beyond brief description about the contents of the volume, with critical reflections on the production of knowledge, research priorities, and factors that contributed to or hindered progress.

The synthesis of the content addressed in the editorials signed by Forattini shows the evolution of the RSP in relation to improvements in the editorial process, the increased rate of article submission, and the intention to give transparency to the process of manuscript evaluation. We emphasize that the increase of papers submitted also led to greater thematic diversity, types of articles, and representativeness of authors from other countries. The context was of growth and development of graduate studies in Brazil and of journal indexing systems, as a means to distinguish the quality of scientific publications, many of them derived from dissertations and theses. This led to increased quality and prestige of the RSP, including the process of peer review and the educational dynamics among reviewers. When celebrating the 30 years anniversary of the RSP in 1996, Forattini indicated prospects of electronic publication, which began two years later with the creation of the Scientific Electronic Library Online (SciELO). Years later, printed volumes were extinct due to the continuous flow of electronic articles (Figure 3 – dark blue).

Other subjects were related to Science (Figure 3 – purple) and Education (Figure 3 – light blue). From the 1990s, he criticized the lack of resources for research, trend of commercialization or scrapping of the public university and lack of priorities in research relevant to the Brazilian reality, reflecting the lack of a national scientific policy. He also criticized the valorization of sophisticated techniques and “cutting-edge technology research”, which led many researchers to leave the country.

The editorials also showed challenges in the field of epidemiology, as socioeconomic issues in the context of globalization emerged, increasing the complexity of public health problems: emergence and re-emergence of diseases, potential pandemics triggered by means of travelers who crossed intercontinental borders in a matter of hours, fears related to bioterrorism and the challenges of the millennium in relation to health and social development, such as poverty eradication, response to the aids epidemic, and others. Forattini had clarity concerning the need to preserve the epidemiologist’s interdisciplinary thinking, rather than constituting teams of experts, so as to increase the effectiveness of epidemiological surveillance (Figure 3 – green).

He also emphasized cases and perspectives in relation to the control of malaria, to the reintroduction of *Aedes aegypti*, to the eradication of polio, and to the importance of studies on insect vectors, in contexts that indicated the risk of re-emergence of diseases (Figure 3 – yellow and orange).

In 1983 Forattini^11^ brings the debate and criticism about the use of the term collective health. At the turn of the century, he pointed out the ethical issues that emerged during the 20th century in the context of social and technological development, indicating them as challenges for the next century (Figure 3 – red).

Furthermore, Forattini was convinced that the national public health issues should be discussed in Portuguese among Brazilian social actors, including scientists trained and operating in the country. Therefore, although recognizing English as the “language of scientific communication”, he argued that Brazilian scientists should publish in Portuguese for their peers and, similarly, for professionals of health services, understanding the role of the RSP in communicating with health workers at all levels.

In many editorials Forattini addressed the crisis in the institutional environment, the difficulties of maintaining the RSP, and the need for external resources, emphasizing in 1981 the importance of the agencies Conselho Nacional de Desenvolvimento Científico e Tecnologico (CNPq) and Fundação de Amparo a Pesquisa do Estado de São Paulo (FAPESP), in addition to the Rectorate of the Universidade de São Paulo (the latter due to having created a specific program to support scientific journals at the university in 1986). CNPq and Capes still support the publication of journals; however, these resources are insufficient to maintain services that would ensure the quality and visibility of the journals.

In 1997, along with nine other journals, the RSP was part of a pilot project for construction of the SciELO program. This was the first collection of Brazilian journals intended for open access, financially and politically supported by FAPESP. Currently, SciELO is present in 14 countries, whose average daily downloads of articles exceeds 1.5 million^14^.

**Figure 4:**
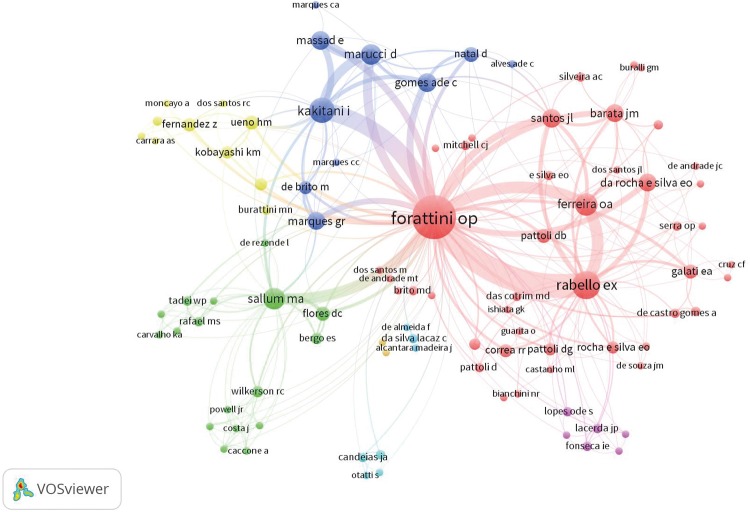
Coauthors network infographic.

Figure 4 shows the coauthors collaboration network concerning Forattini’s published articles.

Coauthors comprise his professors and colleagues, such as Prof. Carlos da Silva Lacaz. The red cluster indicates his publications in medical entomology with Ernesto Xavier Rabello and Dino Pattoli. It also shows the first generation of entomologists trained by him in the Medical Entomology Program, which joined the Faculty of the FSP: Jose Maria Soares Barata, who continued researching *Triatominae*; Eunice Galatti, who specializes in *Phlebotominae*; Almerio de Castro Gomes, Maria Anice Mureb Sallum (Taxonomy), Delsio Natal and Ina Kakitani (Bioecology) continued studies with the *Culicidae* and the Medical Entomology Program, maintaining strong exchanges between the academy and the health services. The green cluster represents the line of research on taxonomy and systematics, which uses molecular biology techniques and that today is led by Sallum. The blue cluster corresponds to the team that worked on the thematic projects and the yellow cluster represents the last generation of researchers trained by Forattini. Most collaborations occurred with Rabello and Kakitani, who were responsible for the fieldwork in his projects.

## CONCLUSIONS

Analysis of the scientific production of Oswaldo Paulo Forattini shows his commitment to public health, in research with vectors, training of researchers, and scientific communication. In this article we showed another equally important contribution, his work as editor of the RSP for 40 of the 50 years of the journal. In all these years, Forattini constantly sought to improve the RSP as to evaluation processes, quality of manuscripts, and visibility of the journal.

## Footnotes

a The collection of articles by Forattini in Zotero is available from: https://www.zotero.org/groups/forattini

b Profile of Oswaldo Forattini at Google Scholar. Available from: https://scholar.google.com.br/citations?hl=pt-BR&user= MAYJRtYAAAAJ
